# Increased Prediction Accuracy in Wheat Breeding Trials Using a Marker × Environment Interaction Genomic Selection Model

**DOI:** 10.1534/g3.114.016097

**Published:** 2015-02-06

**Authors:** Marco Lopez-Cruz, Jose Crossa, David Bonnett, Susanne Dreisigacker, Jesse Poland, Jean-Luc Jannink, Ravi P. Singh, Enrique Autrique, Gustavo de los Campos

**Affiliations:** * Department of Plant, Soil and Microbial Sciences, Michigan State University (MSU), East Lansing, Michigan 4882,; † International Maize and Wheat Improvement Center(CIMMYT), Mexico D.F., Mexico; ‡ Wheat Genetics Resource Center, Department of Plant Pathology and Department of Agronomy, Kansas State University (KSU), 4011 Throckmorton Hall, Manhattan, Kansas 66506; § USDA-ARS and Department of Plant Breeding and Genetics, Cornell University, Ithaca, New York 14853, and; §§ Epidemiology & Biostatistics and Statistics departments, Michigan State University 909 Fee Road, East Lansing, Michigan 48824

**Keywords:** genomic selection, multienvironment, genomic best linear unbiased prediction (GBLUP), marker × environment interaction, International Bread Wheat Screening Nursery, GenPred, shared data resource

## Abstract

Genomic selection (GS) models use genome-wide genetic information to predict genetic values of candidates of selection. Originally, these models were developed without considering genotype × environment interaction(G×E). Several authors have proposed extensions of the single-environment GS model that accommodate G×E using either covariance functions or environmental covariates. In this study, we model G×E using a marker × environment interaction (M×E) GS model; the approach is conceptually simple and can be implemented with existing GS software. We discuss how the model can be implemented by using an explicit regression of phenotypes on markers or using co-variance structures (a genomic best linear unbiased prediction-type model). We used the M×E model to analyze three CIMMYT wheat data sets (W1, W2, and W3), where more than 1000 lines were genotyped using genotyping-by-sequencing and evaluated at CIMMYT’s research station in Ciudad Obregon, Mexico, under simulated environmental conditions that covered different irrigation levels, sowing dates and planting systems. We compared the M×E model with a stratified (*i.e.*, within-environment) analysis and with a standard (across-environment) GS model that assumes that effects are constant across environments (*i.e.*, ignoring G×E). The prediction accuracy of the M×E model was substantially greater of that of an across-environment analysis that ignores G×E. Depending on the prediction problem, the M×E model had either similar or greater levels of prediction accuracy than the stratified analyses. The M×E model decomposes marker effects and genomic values into components that are stable across environments (main effects) and others that are environment-specific (interactions). Therefore, in principle, the interaction model could shed light over which variants have effects that are stable across environments and which ones are responsible for G×E. The data set and the scripts required to reproduce the analysis are publicly available as Supporting Information.

The presence of genotype × environment(G×E) interactions in agricultural experiments usually is expressed as changes in the relative performance of genetic materials across environments; this can manifest as modifications of the ranking of genotypes across environments or simply as changes in the absolute difference in performance between pairs of genotypes. Accounting for G×E has always been a concern in the analysis of multienvironment plant breeding trials, and several models have been proposed and used for describing the mean response of genotypes over environments and for studying and interpreting G×E in agricultural experiments (*e.g.*, Yates and Cochran 1938; Finlay and Wilkinson, 1963; Eberhart and Russell 1966).

The statistical treatment of G×E has evolved over time due to the development of statistical methods and because of changes in the information available, including the increased availability of DNA markers and of precise environmental information. Some approaches deal with G×E implicitly, without explicitly modeling gene × environment interactions; these include some of the early treatment of G×E (*e.g.*, the joint-regression analysis of Yates and Cochran 1938), as well as more modern methods such as the multivariate pedigree- or marker-based models where G×E is modeled using structured or unstructured covariance functions (Piepho, 1997, 1998; Smith *et al.* 2005; Crossa *et al.* 2006; Burgueño *et al.* 2007). These approaches have proved to be effective for exploiting G×E; however, they do not shed light on the underlying basis of G×E (*e.g.*, the relative contribution of different genetic regions to stability and to G×E). When genomic and environmental covariate data are available, G×E can be modeled explicitly by means of marker × environment interactions (M×E). This approach was first used with sparse marker data in quantitative trait loci (QTL) analysis (QTL×E Moreau *et al.* 2004) and also in multilocus models with markers that exhibited “significant” association with the trait of interest (Boer *et al.* 2007). The QTL×E approach has been further extended to multitrait, multienvironment settings (*e.g.*, Malosetti *et al.* 2004, 2008).

Recent developments in genotyping and sequencing technologies have made it possible to use dense genotypic information for genomic selection (GS) (Meuwissen *et al.* 2001). Empirical evidence obtained with plant and animal breeding data has demonstrated that GS can outperform the prediction accuracy of pedigree-based methods or that of models based on a reduced number of loci (de los Campos *et al.* 2009, 2010; Crossa *et al.* 2010, 2011; Heslot *et al.* 2012; Pérez-Rodríguez *et al.* 2012). This has prompted the relatively fast adoption of GS in plant and animal breeding. GS models originally were developed for a single trait evaluated in a single environment, and most analyses published so far are based on within-environment analyses.

Recently, several studies have proposed using GS models that accommodate G×E. For instance, Burgueño *et al.*(2012) extended the single-trait, single-environment genomic best linear unbiased prediction(GBLUP) model to a multienvironment context and reported important gains in prediction accuracy with the multienvironment model relative to single-environment analysis. More recently, Heslot *et al.* (2014) and Jarquin *et al.* (2014) considered modeling G×E using both genetic markers and environmental covariates. These studies also showed that modeling G×E can give substantial gains in prediction accuracy.

Following ideas originally used for QTL analysis in multienvironment trials(Van Eeuwijk *et al.* 2005; Boer *et al.* 2007; Malosetti *et al.* 2004, 2008), we present GS models that accommodate G×E by explicitly modeling interactions between all available markers and environments. Relative to multivariate approaches where G×E is modeled using covariance parameters(*e.g.*, Smith *et al.* 2005), the M×E approach has advantages and disadvantages. First, the M×E models presented here can be easily implemented using existing software for GS. Second, the model can be implemented using both shrinkage methods as well as variable selection methods. Third, the M×E model decomposes effects into components that are common across environments (stability) and environment-specific deviations; this information, which is not provided by standard multienvironment mixed models, can be used to identify genomic regions whose effects are stable across environments and others that are responsible for G×E. On the other hand, the M×E model imposes restrictions on the patterns of G×E, and, for reasons that we discuss in this article, the model is best suited for the joint analysis of positively correlated environments.

In this study, we applied the M×E model to extensive data sets where wheat lines were evaluated under contrasting environmental conditions in replicated field trials. This allowed us to identify under which conditions the M×E model is most effective. We show theoretically and demonstrate empirically that the magnitude of the main and interaction variance is directly related to the phenotypic correlation between environments and that the M×E model performs best when the set of environments analyzed showed positive and similar correlations. Indeed, when the set of environments analyzed had moderate or high positive correlations, the M×E model yielded substantial gains in prediction accuracy relative to an across-environment GS model that assumes homogeneity of effects across environments, and, depending on the prediction problem, it either performed similarly or outperformed the stratified (*i.e.*, within-environment) analyses. In the rest of this article, we describe the methods used and present empirical results obtained when the M×E model was applied to three wheat data sets. We also provide, as online materials, scripts that implement the interaction models using the BGLR R-package (de los Campos and Pérez-Rodriguez 2014).

## Materials and Methods

The data used in this study are from CIMMYT’s Global Wheat Program and consist of a set of wheat lines evaluated under managed environmental conditions; these conditions were designed to simulate target mega-environments. The wheat lines included in this study were later part of the 45th, 46th, and 47th International Bread Wheat Screening Nurseries and distributed worldwide.

Three files containing phenotypic and genotypic information on the three data sets used in this study (45th, 46th and 47th International Bread Wheat Screening Nurseries) are provided as Supporting Information, File S1, File S2, and File S3, respectively.

### Phenotypic data

The phenotypic data consisted of adjusted grain yield(ton/ha) records collected during three evaluation cycles (W1: cycle 2010−2011, N = 732; W2: cycle 2011−2012, N = 672; and W3: cycle 2012−2013, N = 811); each cycle included a different set of advanced breeding lines. All trials were established at CIMMYT’s main wheat breeding station at Cd. Obregon, Mexico. The experimental design was an alpha-lattice with three replicates per line and environment. Wheat lines were evaluated under three irrigation regimes(2i = two irrigations giving moderate drought stress, 5i = five irrigations representing an optimally irrigated crop, and 0i = no irrigation or drip irrigation, representing high drought stress), two planting systems (B = bed planting; F = planting on the flat) and two planting dates (N = normal and H = late, simulating heat at the grain-filling stage). In the 2i and 5i regimes, irrigation was applied without measuring soil moisture, and each irrigation added 100 mm of water. Some of the trials were managed using no-tillage(hereinafter denoted as Z). Table 1 gives the number of phenotypic records per simulated environment and cycle. The phenotype used in the analysis was the best linear unbiased estimate of grain yield obtained from a linear model applied to the alpha-lattice design of each cycle-environment combination.

**Table 1:** Number of phenotypic records per cycle (W1, W2, and W3) and environment

Environment*^a^*	Data Set		
	W1 (Cycle 2010-11, 45th IBWSN)	W2 (Cycle 2011-12, 46th IBWSN)	W3 (Cycle 2012-13, 47th IBWSN)
0iBN	693	–	–
0iFN	–	670	807
2iBN	693	–	807
5iBNZ	693	–	–
5iBH	–	670	807
5iBN	–	670	807
5iFN	693	670	807
Total	2772	2680	4035

a Environments are described by a sequence of codes: 0i, 2i, and 5i denote the number of irrigation cycles; B/F denotes whether the planting system was ‘bed’ (B) or ‘flat’ (F); N/H denotes whether planting date was normal (N) or late (H, simulating heat); Z indicates no tillage. IBWSN denotes International Bread Wheat Screening Nurseries

### Genotypic data

Genotypes were derived using genotyping-by-sequencing technology (GBS; Poland *et al.* 2012). GBS markers with a minor allele frequency lower than 0.05 were removed. As is typical of GBS genotypes, all markers had a high incidence of uncalled genotypes. In our quality control pipeline, we applied thresholds for incidence of missing values aimed at maintaining relatively large and similar numbers of markers per data set. To this end, we removed markers with more than 60%(W1 and W2) or 80% (W3) missing values; this left 15,744 (W1 and W2) and 14,217(W3) GBS markers available for analysis. Finally, only lines with more than 2000 called GBS markers were used in the data analysis; this left 693 (W1), 670 (W2), and 807(W3) lines.

### Statistical models

For each evaluation cycle (W1, W2, and W3), we considered three approaches: (i) a stratified analysis obtained by regressing phenotypes on markers separately in each environment (we refer to this approach, indistinctively, as to single-environment, within-environment or stratified analysis); (ii) a combined analysis based on a GS model where marker effects are assumed to be constant across environments (*i.e.*, ignoring M×E) (hereinafter referred to as the “across-environment” model); and (iii) using a M×E model that allows analyzing data from multiple environments jointly and accounts for G×E. Each of these approaches is discussed in the sections to follow.

#### Stratified analysis:

This model is obtained by regressing the phenotype vector containing the records available in the *j^th^* environment, $y_{j}=\left\{y_{ij}\right\}$, where *i* indexes lines (individuals) and *j* indexes environments, on markers using a linear model in the form:$\;y_{ij}=\mu_{j}+\sum_{k=1}^{p}x_{ijk}\beta_{jk}+\;\varepsilon_{ij}$, (*i* = 1,2,…,*n* individuals; *j* = 1,2,…*s* environments; *k* = 1,2,…,*p* markers) or, in matrix notation,

$$y_{j}=1\mu_{j}+X_{j}\beta_{j}+\varepsilon_{j}$$ (1a)

where $\mu_{j}$ is an intercept, $X_{j}=\{x_{ijk}\}$ is a matrix of marker-centered and standardized genotypes(*i.e.*, each marker was centered by subtracting the mean and standardized by dividing by the sample standard deviation), $\beta_{j}=\{\beta_{jk}\}$ is a vector of marker effects and $\varepsilon_{j}$ is a vector of model residuals. Note that, in a full-factorial design where all lines are evaluated in all environments, $X_{1}=X_{2}=\ldots =X_{s}$. Following the standard assumptions of the GBLUP model (*e.g.*, Vanraden, 2007, 2008), marker effects and model residuals were assumed to be independent of each other and both normally distributed: $\beta_{j}∼N(0,I\sigma_{\beta_{j}}^{2})$, and $\varepsilon_{j}∼N\left(0,I\sigma_{\varepsilon_{j}}^{2}\right)$. Setting $u_{j}=X_{j}\beta_{j},$ we have that the aforementioned model also can be represented as follows:

$$y_{j}=1\mu_{j}+u_{j}+\varepsilon_{j}$$ (1b)

with $u_{j}∼N\left(0,G_{j}\sigma_{u_{j}}^{2}\right)$, where $G_{j}=\frac{X_{j}X_{j}{}^{\prime }}{p}\text{ was }$obtained using the cross-product of (centered and standarized) marker genotypes and scaled by dividing by the number of markers. Because all markers were standardized to a unit variance, this gives an average diagonal value of $G_{j}$ equal to one.

Box 1a in File S4 provides an R-script that implements the single-environment model described previously using the BGLR R-package (de los Campos and Pérez-Rodriguez 2014).

#### Across-environment GBLUP model:

another approach consists of assuming that effects of markers are the same across environments, that is: $\beta_{1}=\beta_{2}=\ldots =\beta_{s}=\beta$; therefore the regression model (1b) becomes (assuming s = 3, for ease of notation):

$$\left[\begin{array}{c} y_{1}\\ y_{2}\\ y_{3} \end{array}\right]=\left[\begin{array}{c} 1\mu_{1}\\ 1\mu_{2}\\ 1\mu_{3} \end{array}\right]+\left[\begin{array}{c} X_{1}\\ X_{2}\\ X_{3} \end{array}\right]\beta +\left[\begin{array}{c} \varepsilon_{1}\\ \varepsilon_{2}\\ \varepsilon_{3} \end{array}\right]$$ (2a)

In a GBLUP-context, one will assume $\beta ∼N(0,I\sigma_{\beta }^{2})$ and the aforementioned model can be represented as a random effect model as follows:

$$\left[\begin{array}{c} y_{1}\\ y_{2}\\ y_{3} \end{array}\right]=\left[\begin{array}{c} 1\mu_{1}\\ 1\mu_{2}\\ 1\mu_{3} \end{array}\right]+\left[\begin{array}{c} u_{1}\\ u_{2}\\ u_{3} \end{array}\right]+\left[\begin{array}{c} \varepsilon_{1}\\ \varepsilon_{2}\\ \varepsilon_{3} \end{array}\right]$$ (2b)

where $u_{j}=X_{j}\beta$, and $u={\left(u^{\prime }{}_{1},u^{\prime }{}_{2},\;u^{\prime }{}_{3}\right)}^{\prime }∼N\left(0,G_{0}\sigma_{u}^{2}\right)$, where

$$G_{0}=\left[\begin{array}{ccc} X_{1}X_{1}{}^{\prime } & X_{1}X_{2}{}^{\prime } & X_{1}X_{3}{}^{\prime }\\ X_{2}X_{1}{}^{\prime } & X_{2}X_{2}{}^{\prime } & X_{2}X_{3}{}^{\prime }\\ X_{3}X_{1}{}^{\prime } & X_{3}X_{2}{}^{\prime } & X_{3}X_{3}{}^{\prime } \end{array}\right]/p$$

is a marker-derived genomic relationship matrix. It is worth noting that, for balanced data, the model of expression (2a) is equivalent to fitting a genomic regression model using the average performance of each line across environments as a phenotype.

Box 2a in File S4 provides an R-script that implements the across-environment model described above using the BGLR R-package (de los Campos and Pérez-Rodriguez 2014).

#### M×E GBLUP model:

In the model in expression (1a), marker effects ($\beta_{j}$) are estimated separately for each environment; therefore, in this model there is no borrowing of information across environments. On the other hand, in the model in expression (2a), all data are used to estimate marker effects; however, in that model borrowing of information is achieved by assuming that effects are constant across environments. We now consider an interaction model that aims at benefiting from borrowing information across environments while allowing marker effects to change across environments. In the M×E model, the effect of the *k^th^* marker on the *j^th^* environment ($\beta_{jk}$) is described as the sum of an effect common to all environments ($b_{0k}$), plus a random deviation ($b_{jk}$) peculiar to the *j^th^* environment, that is $\beta_{jk}=b_{0k}+b_{jk}$. Therefore, the equation for data from the *j^th^* environment becomes $y_{ij}=\mu_{j}+\sum_{k=1}^{p}x_{ijk}\left(b_{0k}+b_{jk}\right)+\varepsilon_{ij}$, or, in matrix notation and assuming, for ease of notation, only three environments,

$$\left[\begin{array}{c} y_{1}\\ y_{2}\\ y_{3} \end{array}\right]=\left[\begin{array}{c} 1\mu_{1}\\ 1\mu_{2}\\ 1\mu_{3} \end{array}\right]+\left[\begin{array}{c} X_{1}\\ X_{2}\\ X_{3} \end{array}\right]b_{0}+\left[\begin{array}{ccc} X_{1} & 0 & 0\\ 0 & X_{2} & 0\\ 0 & 0 & X_{3} \end{array}\right]\left[\begin{array}{c} b_{1}\\ b_{2}\\ b_{3} \end{array}\right]+\left[\begin{array}{c} \varepsilon_{1}\\ \varepsilon_{2}\\ \varepsilon_{3} \end{array}\right],$$ (3a)

where the vectors of main and interaction effects and model residuals were all assumed to be normally distributed, specifically: $b_{0}∼N\left(0,I\sigma_{b_{0}}^{2}\right)$, $b_{j}∼N\left(0,I\sigma_{b_{j}}^{2}\right)$ and $\varepsilon_{j}∼N\left(0,I\sigma_{\varepsilon }^{2}\right)$. The aforementioned model can be represented as a two-variance component GBLUP model, specifically, letting $y=\left(y_{1}^{\prime },y_{2}^{\prime },y_{3}^{\prime }\right)\prime$,

$$\mu =\left[\begin{array}{c} 1\mu_{1}\\ 1\mu_{2}\\ 1\mu_{3} \end{array}\right],u_{0}=\left[\begin{array}{c} X_{1}\\ X_{2}\\ X_{3} \end{array}\right]b_{0},u_{1}=\left[\begin{array}{ccc} X_{1} & 0 & 0\\ 0 & X_{2} & 0\\ 0 & 0 & X_{3} \end{array}\right]\left[\begin{array}{c} b_{1}\\ b_{2}\\ b_{3} \end{array}\right]$$

the model can be represented as

$$y=\mu +u_{0}+u_{1}+\varepsilon$$, (3b)

where $u_{0}∼N(0,\sigma_{u0}^{2}G_{0})$, $u_{1}∼N(0,G_{1})$, $\varepsilon ∼N(0,I\sigma_{\varepsilon }^{2})$ where $G_{0}$ is as described previously and

$$G_{1}=\left[\begin{array}{ccc} \sigma_{u1}^{2}X_{1}X_{1}{}^{\prime } & 0 & 0\\ 0 & \sigma_{u2}^{2}X_{2}X_{2}{}^{\prime } & 0\\ 0 & 0 & \sigma_{u3}^{2}X_{3}X_{3}{}^{\prime } \end{array}\right]/p$$

In this model, the main effect ($u_{0}$) allows borrowing information between environments (through the off-diagonal blocks of $G_{0}$) and $u_{1}$ captures environment-specific effects. The relative importance of these two terms is determined by the corresponding variance components that are inferred from the data.

Box 3a in File S4 provides an R-script that implements the M×E model described previously using the BGLR R-package (de los Campos and Pérez-Rodriguez 2014).

### Software

The aforementioned models can be implemented using standard software for GS. For our implementation, we used the R (R Core Team 2013) package Bayesian generalized linear regression (BGLR, de los Campos and Pérez-Rodriguez 2014). This software does not allow fitting group-specific error variances; therefore, in our interaction model and in our across-environment analyses, we fit the models assuming homogeneous error variances across environments. The code used to implement these models is provided in the Supporting Information; technical details and several examples of the use of the package for genome-enabled prediction can be found in de los Campos and Pérez-Rodriguez (2014). We used a Bayesian model assuming Gaussian priors for the marker effects. The BGLR package assigns scaled-inverse χ^2^ densities to the variance parameters whose hyperparameters were given values using the default rules implemented in BGLR, which assign 5 degrees of freedom and calculates the scale parameter based on the sample variance of the phenotypes. Further details are given in de los Campos and Pérez-Rodriguez (2014).

### Statistical analysis

The aforementioned models were fitted to data from each of the cycles (W1−W3), separately. For each cycle data we performed analysis: (i) within-environment (see expression 1a), or (ii) by pairs of environments or, (iii) using data from all environments together. These last two approaches were implemented either using the model in expression (2a) or the interaction model expression (3a).

Models were fitted to each of the full data sets to derive estimates of variance components. Subsequently, we assessed prediction accuracy using training-testing (*i.e.*, TRN-TST) random partitions (see below). For this validation procedure, all the parameters of the models, including variance components, were re-estimated from TRN data in each of the TRN-TST partitions. In all cases, inferences and predictions were based on 55,000 samples collected from the posterior distribution after discarding 5000 samples for burn-in.

Prediction accuracy was assessed using 50 TRN-TST random partitions; we used this approach because with a replicated TRN-TST design one can obtain as many partitions as one needs and this allows estimating SEs of estimates of prediction accuracy more precisely than with a cross-validation approach. Following Burgueño *et al.* (2012), we considered two different prediction problems. First (CV1), we assessed prediction accuracy of the models when TRN and TST data consist of disjoint sets of lines; this approach mimics the prediction problem faced by breeders when lines have not been evaluated in any field trials. To generate TRN and TST sets in CV1 we simply assigned completely at random 70% of the lines to TRN and the remaining 30% to TST.

Second (CV2), we considered the problem of predicting the performance of lines in environments in which lines have not been evaluated. This validation design mimics the prediction problem faced by breeders in incomplete field trials where lines are evaluated in some but not all target environments. TRN-TST partitions for CV2 were obtained as follows: in each data set (*w = 1,2,3*), each line (*i = 1,…*,$\;n_{w}$) had records in $s_{w}$ environments (*j = 1,…*,$\;s_{w},\;s_{w}=4$ in W1 and W2, and $s_{w}=5$ in W3); therefore, the total number of records available per data set was $N_{w}=n_{w}\times s_{w}$. To select the entries in the TST data set, we first chose $0.3\times N_{w}$ IDs (*i.e.*, lines) at random and subsequently randomly picked one environment per line from the index *j = 1,…*,$\;s_{w}$. The resulting cells (ij) were assigned to the TST data set, and the ones not selected through this algorithm were used for model TRN. Lines were sampled without replacement if $n_{w}\geq 0.3\times N_{w}$ and with replacement otherwise. Boxes 4a and 4b in File S4 provide the R-code used to generate TRN-TST partitions in CV1 and CV2, and Boxes 5 and 6 illustrate how to fit models and evaluate prediction accuracy for a TRN-TST partition.

For each TRN-TST partition, models were fitted to the TRN data set and prediction accuracy was assessed by computing Pearson’s product-moment correlation between predictions and phenotypes in the TST data set, within environment. The same TRN-TST partitions were used to assess the prediction accuracy of each of the models; this yielded 50 correlation estimates for each model, data set, and we report the average correlation (across partitions) and SEs.

## Results

**Figure 1:**
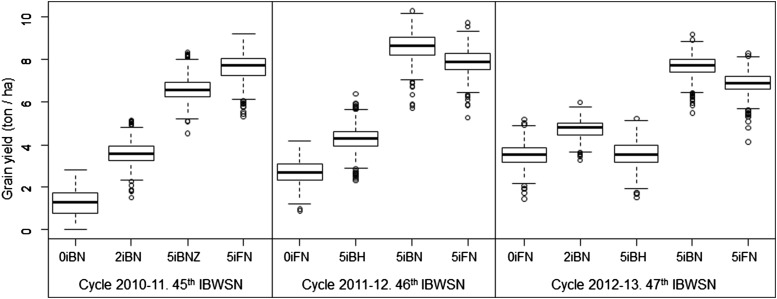
Box-plot of adjusted grain yield by environment and cycle. Environments are denoted by a sequence of codes: 0i, 2i, and 5i denote the number of irrigation cycles; B/F denotes whether the planting system was “bed” (B) or “flat” (F); N/H denotes whether planting date was normal (N) or late (H, simulating heat); Z indicates no tillage.

Figure 1 shows box-plots of adjusted yield per data set and environmental condition. As expected, average yield increased with the number of irrigation events and, other factors being equal, late planting (H) produced lower yields than normal planting (N). In all cases, the empirical distribution of grain yield within data set and environment was reasonably symmetric.

Table 2 gives the SDs of grain yield for each environment-cycle combination and the (empirical) phenotypic correlations of adjusted grain yield across environments, within cycle. The average SD of grain yield was rather stable across environments, ranging from 0.41 to 0.65. In the first cycle (W1), correlations across environments were moderately positive, ranging from 0.22 to 0.53 and, as one would expect, environments with similar irrigation levels (*e.g.*, 0i and 2i) exhibited greater correlations than environments with very different numbers of irrigations (*e.g.*, 0i *vs.* 5i). In the second evaluation cycle (W2), three environments received five irrigations (5i) and one received none (0i). The correlations among environments with five irrigations were also moderately positive, ranging from 0.33 to 0.41. However, the correlation between drought environments (0i) and environments having five irrigations was almost zero (−0.05). Finally, in the third evaluation cycle (W3), the two environments with five irrigations and normal planting dates (5iBN and 5iFN) were positively correlated (0.55); however, environments with different irrigation levels or different planting dates (N/H) showed very low, and even negative, correlations. Interestingly, environments 0iFN and 5iBH, which differ across all factors, showed a moderate sample correlation (0.30).

**Table 2:** SD (diagonal) and sample correlation (lower-triangular) between grain yields evaluated under different environmental conditions, by cycle

	0iBN	2iBN	5iBNZ	5iFN	−
W1 (cycle 2010−2011, 45th IBWSN)					
Environment *^a^*					
0iBN	0.61	−	−	−	−
2iBN	0.53 (0.47−0.58)	0.51	−	−	−
5iBNZ	0.25 (0.18−0.32)	0.34 (0.27−0.40)	0.60	−	−
5iFN	0.26 (0.19 −0.33)	0.33 (0.26−0.39)	0.22 (0.15−0.29)	0.63	−

W2 (cycle 2011−2012, 46th IBWSN)					
Environment	0iFN	5iBH	5iBN	5iFN	−
0iFN	0.54	−	−	−	−
5iBH	0.34 (0.27−0.41)	0.60	−	−	−
5iBN	−0.05 (-0.13,0.02)	0.33 (0.26,0.39)	0.65	−	−
5iFN	0.31 (0.24−0.38)	0.41 (0.35,0.47)	0.41 (0.35−0.47)	0.58	−

W3 (cycle 2012−2013, 47th IBWSN)					
Environment	0iFN	2iBN	5iBH	5iBN	5iFN
0iFN	0.51	−	−	−	−
2iBN	0.17 (0.10−0.23)	0.41	−	−	−
5iBH	0.30 (0.24−0.36)	−0.03 (-0.10, 0.04)	0.60	−	−
5iBN	−0.10 (−0.16, −0.03)	0.12 (0.05−0.19)	−0.09 (−0.16, −0.02)	0.49	−
5iFN	−0.01 (−0.08, 0.06)	0.04 (−0.03, 0.10)	0.02 (−0.05, 0.09)	0.55 (0.50−0.59)	0.51

95% confidence interval for the correlations are given in parentheses. IBWSN, International Bread Wheat Screening Nurseries

a Environments are described by a sequence of codes: 0i, 2i, and 5i denote the number of irrigation cycles; B/F denotes whether the planting system was “bed” (B) or “flat” (F); N/H denotes whether planting date was normal (N) or late (H, simulating heat); Z indicates no tillage.

### Estimates of variance components

Table 3, Table 4, and Table 5 provide estimates of variance components per model and environment for cycles W1, W2, and W3, respectively. The estimates reported in these tables correspond to the full-data analyses.

**Table 3:** Estimates of variance components (estimated posterior SD) by model and environment, cycle 2010−2011 (W1)

Models/Environments*^a^*		Residual		Main Effect		Interaction (M×E)	R-Squared*^b^*	
Single environment								
0iBN		0.529(0.037)		0.400(0.066)		−	0.429(0.049)	
2iBN		0.362(0.031)		0.609(0.082)		−	0.624(0.044)	
5iBNZ		0.549(0.043)		0.533(0.089)		−	0.489(0.053)	
5iFN		0.584(0.041)		0.370(0.064)		−	0.386(0.050)	
		Interaction Model	Across-Env Model	Interaction Model	Across-Env Model	Interaction Model	Interaction Model	Across-Env Model
Pairs of environments								
0iBN	Cor = 0.53	0.429(0.022)	0.497(0.023)	0.427(0.057)	0.433(0.054)	0.092(0.031)	0.545(0.036)	0.464(0.036)
2iBN						0.118(0.039)	0.557(0.035)	
0iBN	Cor = 0.25	0.527(0.029)	0.714(0.032)	0.188(0.054)	0.239(0.042)	0.220(0.061)	0.433(0.046)	0.250(0.036)
5iBNZ						0.369(0.084)	0.510(0.048)	
0iBN	Cor = 0.26	0.558(0.028)	0.681(0.029)	0.173(0.045)	0.228(0.037)	0.198(0.055)	0.396(0.044)	0.250(0.033)
5iFN						0.196(0.060)	0.395(0.046)	
2iBN	Cor = 0.34	0.434(0.025)	0.609(0.028)	0.367(0.062)	0.374(0.052)	0.202(0.059)	0.564(0.039)	0.379(0.037)
5iBNZ						0.309(0.081)	0.605(0.043)	
2iBN	Cor = 0.33	0.474(0.026)	0.634(0.028)	0.254(0.052)	0.290(0.042)	0.252(0.061)	0.513(0.041)	0.313(0.034)
5iFN						0.191(0.060)	0.481(0.046)	
5iBNZ	Cor = 0.22	0.577(0.031)	0.723(0.031)	0.134(0.046)	0.221(0.037)	0.351(0.084)	0.453(0.049)	0.234(0.032)
5iFN						0.215(0.067)	0.374(0.048)	
All environments								
0iBN		0.485(0.018)	0.681(0.020)	0.316(0.042)	0.272(0.034)	0.116(0.038)	0.472(0.035)	0.285(0.027)
2iBN						0.117(0.036)	0.471(0.032)	
5iBNZ						0.396(0.073)	0.594(0.034)	
5iFN						0.263(0.059)	0.543(0.037)	

a Environments are described by a sequence of codes: 0i, 2i, and 5i denote the number of irrigation cycles; B/F denotes whether the planting system was “bed” (B) or “flat”(F); N/H denotes whether planting date was normal (N) or late (H, simulating heat); Z indicates no tillage.

b Model *R*^2^ was computed as the ratio of the sum of the main and interaction variance, relative to the total variance (residual + main effect + interaction). Env, environment.

**Table 4:** Estimates of variance components (estimated posterior SD) by model and environment, cycle 2011−2012 (W2)

Models/Environments*^a^*		Residual		Main Effect		Interaction (M×E)	R-Squared*^b^*	
Single environment								
0iFN		0.563(0.046)		0.512(0.099)		−	0.473(0.060)	
5iBH		0.606(0.046)		0.488(0.088)		−	0.443(0.055)	
5iBN		0.492(0.041)		0.612(0.097)		−	0.551(0.052)	
5iFN		0.611(0.046)		0.423(0.080)		−	0.407(0.055)	
Pairs of environments		Interaction Model	Across-Env Model	Interaction Model	Across-Env Model	Interaction Model	Interaction Model	Across-Env Model
0iFN	Cor = 0.34	0.541(0.032)	0.712(0.034)	0.371(0.079)	0.344(0.060)	0.223(0.073)	0.520(0.051)	0.325(0.043)
5iBH						0.243(0.073)	0.528(0.049)	
0iFN	Cor =-0.05	0.532(0.032)	0.841(0.036)	0.056(0.023)	0.149(0.029)	0.476(0.096)	0.495(0.053)	0.151(0.026)
5iBN						0.500(0.091)	0.506(0.048)	
0iFN	Cor = 0.31	0.532(0.031)	0.729(0.035)	0.389(0.082)	0.324(0.058)	0.232(0.071)	0.535(0.050)	0.307(0.042)
5iFN						0.199(0.063)	0.521(0.049)	
5iBH	Cor = 0.33	0.537(0.029)	0.668(0.031)	0.370(0.065)	0.388(0.057)	0.184(0.062)	0.505(0.044)	0.367(0.039)
5iBN						0.199(0.062)	0.512(0.042)	
5iBH	Cor = 0.41	0.553(0.030)	0.651(0.032)	0.441(0.075)	0.428(0.067)	0.147(0.050)	0.513(0.044)	0.396(0.042)
5iFN						0.125(0.043)	0.503(0.044)	
5iBN	Cor = 0.41	0.539(0.029)	0.617(0.030)	0.381(0.065)	0.407(0.059)	0.169(0.058)	0.502(0.044)	0.396(0.040)
5iFN						0.130(0.047)	0.484(0.043)	
All environments								
0iFN		0.519(0.021)	0.737(0.022)	0.436(0.056)	0.334(0.045)	0.400(0.076)	0.614(0.034)	0.311(0.031)
5iBH						0.147(0.046)	0.528(0.036)	
5iBN						0.266(0.069)	0.572(0.037)	
5iFN						0.097(0.033)	0.504(0.034)	

a Environments are described by a sequence of codes: 0i, 2i, and 5i denote the number of irrigation cycles; B/F denotes whether the planting system was “bed” (B) or “flat” (F); N/H denotes whether planting date was normal (N) or late (H, simulating heat); Z indicates no tillage.

b Model *R*^2^ was computed as the ratio of the sum of the main and interaction variance, relative to the total variance (residual + main effect + interaction).

**Table 5:** Estimates of variance components (estimated posterior standard deviation) by model and environment, cycle 2012-13 (W3)

Models/Environments*^a^*		Residual		Main Effect		Interaction (M×E)	R-Squared*^b^*	
Single environment								
0iFN		0.296(0.052)		0.674(0.087)		−	0.692(0.061)	
2iBN		0.410(0.059)		0.585(0.091)		−	0.589(0.068)	
5iBH		0.220(0.037)		0.653(0.067)		−	0.745(0.047)	
5iBN		0.386(0.068)		0.674(0.105)		−	0.633(0.073)	
5iFN		0.491(0.071)		0.563(0.103)		−	0.532(0.077)	
Pairs of environments		Interaction Model	Across-Env Model	Interaction Model	Across-Env Model	Interaction Model	Interaction Model	Across-Env Model
0iFN	Cor = 0.17	0.336(0.042)	0.758(0.031)	0.204(0.040)	0.214(0.031)	0.413(0.070)	0.645(0.051)	0.220(0.027)
2iBN						0.470(0.080)	0.664(0.051)	
0iFN	Cor = 0.30	0.243(0.033)	0.622(0.026)	0.193(0.038)	0.259(0.029)	0.540(0.076)	0.750(0.041)	0.295(0.026)
5iBH						0.426(0.065)	0.717(0.043)	
0iFN	Cor=-0.10	0.328(0.046)	0.898(0.034)	0.045(0.015)	0.095(0.019)	0.588(0.079)	0.657(0.055)	0.096(0.018)
5iBN						0.709(0.093)	0.694(0.052)	
0iFN	Cor=-0.01	0.373(0.047)	0.872(0.034)	0.066(0.024)	0.118(0.023)	0.512(0.076)	0.606(0.056)	0.119(0.022)
5iFN						0.647(0.096)	0.654(0.055)	
2iBN	Cor=-0.03	0.279(0.037)	0.837(0.032)	0.051(0.017)	0.126(0.021)	0.712(0.086)	0.730(0.045)	0.131(0.020)
5iBH						0.534(0.063)	0.676(0.047)	
2iBN	Cor = 0.12	0.417(0.051)	0.786(0.032)	0.089(0.030)	0.171(0.027)	0.473(0.086)	0.570(0.062)	0.179(0.025)
5iBN						0.531(0.093)	0.594(0.060)	
2iBN	Cor = 0.04	0.462(0.050)	0.854(0.034)	0.072(0.025)	0.135(0.025)	0.442(0.079)	0.524(0.060)	0.137(0.023)
5iFN						0.514(0.090)	0.556(0.060)	
5iBH	Cor=-0.09	0.253(0.038)	0.877(0.033)	0.045(0.015)	0.103(0.019)	0.568(0.066)	0.707(0.049)	0.106(0.018)
5iBN						0.825(0.089)	0.773(0.041)	
5iBH	Cor = 0.02	0.282(0.041)	0.838(0.033)	0.081(0.028)	0.135(0.023)	0.497(0.067)	0.671(0.051)	0.140(0.022)
5iFN						0.777(0.096)	0.751(0.045)	
5iBN	Cor = 0.55	0.288(0.031)	0.463(0.023)	0.678(0.055)	0.617(0.052)	0.159(0.046)	0.741(0.033)	0.571(0.027)
5iFN						0.188(0.051)	0.748(0.033)	
All environments								
0iFN		0.310(0.030)	0.886(0.021)	0.113(0.018)	0.101(0.014)	0.539(0.064)	0.676(0.039)	0.102(0.014)
2iBN						0.614(0.075)	0.699(0.039)	
5iBH						0.475(0.054)	0.653(0.038)	
5iBN						0.639(0.075)	0.706(0.038)	
5iFN						0.637(0.079)	0.706(0.040)	

a Environments are described by a sequence of codes: 0i, 2i, and 5i denote the number of irrigation cycles; B/F denotes whether the planting system was “bed” (B) or “flat” (F); N/H denotes whether planting date was normal (N) or late (H, simulating heat); Z indicates no tillage.

b Model *R*^2^ was computed as the ratio of the sum of the main and interaction variance, relative to the total variance (residual + main effect + interaction).

#### Stratified analysis:

The proportion of variance explained by the regression on markers (R^2^ computed based on estimates of variance components) estimated from the stratified (single-environment) analysis ranged from moderate (∼0.4) to high (∼0.7); in general, models fitted data better in W3 (Table 5) than in W1 or W2.

#### Across-environment model:

The estimated residual variance of the across-environment model was typically larger (and consequently the R^2^ was lower) than that of the interaction model indicating that a sizable proportion of the G×E went to the residual of the across-environment model.

#### M×E model:

In the interaction models, the total genomic variance can be partitioned into a main effect and an interaction component. This partition showed that the relative importance of the main effect was greater when the environments analyzed jointly were positively correlated. On the other hand, as one would expect, when the environments analyzed jointly had low correlations, the estimated interaction variance was greater. For instance, in W1, the analysis of pairs of environments exhibiting sample phenotypic correlations smaller than 0.3 (0iBN + 5iBNZ, 0iBN + 5iFN, and 5iBNZ + 5iFN) yielded estimates of variance components in the M×E model where the main effect explained less than 50% of the total genomic variance, computed as the sum of the main effect plus interaction variance estimates (see Table 3). On the other hand, the pairs of environments showing correlations larger than 0.3 (2iBN + 5iBNZ and 2iBN + 5iFN) gave estimates of variance components where the main effect explained between 50 and 70% of the genomic variance. Finally, in W1, the pair of environments with the largest sample phenotypic correlation (0iBN + 2iBN) had estimates of variance components such that the main effect explained about 80% of the total genomic variance.

Similar patterns were observed in W2 and W3. Indeed, in W2, the main effects of markers explained more than 60% of the genomic variance for pairs of environments having sample phenotypic correlations greater than 0.33 (Table 4); on the other hand, in the two environments showing a low correlation (0iFN + 5iBN), the main effect explained only about 10% of the genomic variance. In W3 data set, pairs of environments with sample phenotypic correlations smaller than 0.1 had an estimated proportion of genomic variance explained by main effects that was smaller than 0.2. At the other extreme, for the pair of environments showing the greatest correlation (5iBN + 5iFN), the proportion of variance explained by main effects was close to 0.8 (Table 5). Finally, as expected, in the joint analysis of all environments, the variance of the main effect was largest in W1 and W2 (0.316 and 0.436, respectively), where several pairs of environments had sample phenotypic correlations that were moderately high, and considerably smaller in W3 (0.113), where many pairs of environments had phenotypic correlations that were negative or close to zero.

### Assessment of prediction accuracy

**Figure 2:**
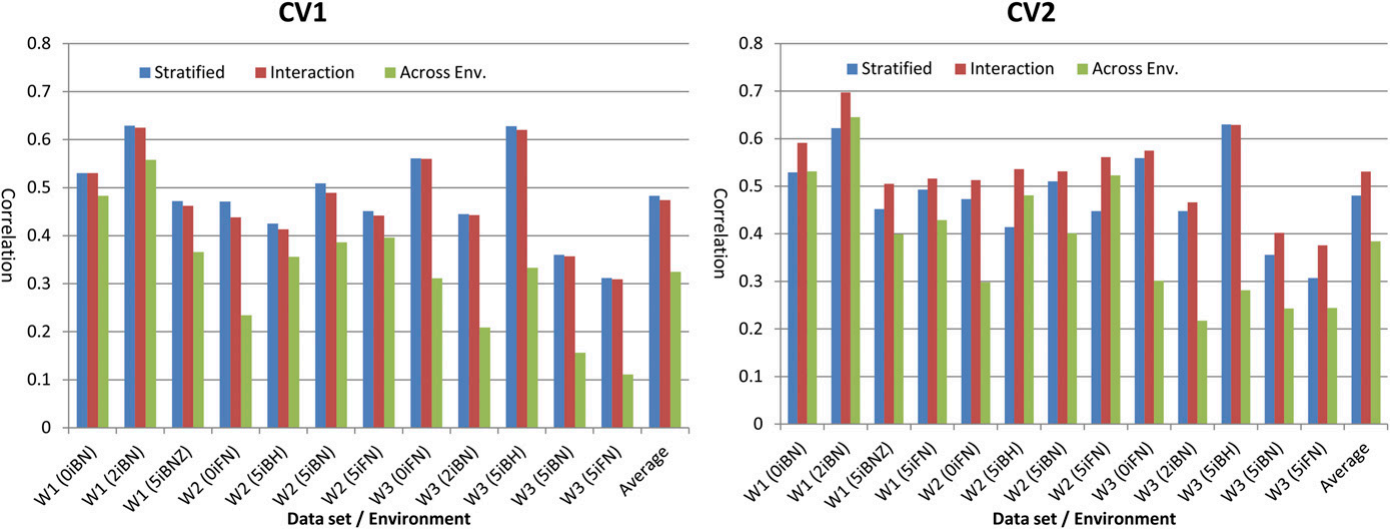
Correlation between phenotypes and predictions (average over 50 TRN-TST partitions) by model (stratified, interaction, and across-environment analysis), validation design (CV1, left, CV2 right), data set and simulated environment (horizontal axis). TRN-TST, training-testing.

The average correlation and the estimated SD (both computed using 50 TRN-TST partitions, each with 70% of records in the TRN data set and 30% in TST data set) obtained in CV1 are reported in Table 6, Table 7, and Table 8 and those obtained in CV2 are reported in Table 9, Table 10, and Table 11. A summary of these results is given in Figure 2. As one would expect, the levels of prediction accuracy (correlation) were slightly greater in CV2 than in CV1. In CV1, the stratified analysis and the interaction model performed similarly (average correlation of 0.48 and 0.47 for the stratified and interaction models, respectively), and the across-environment analysis was the worst one (the average correlation in CV1 was 0.33, that is about 30% lower correlation than the stratified analysis or the interaction model). On the other hand, in CV2, the interaction model gave the greatest levels of prediction accuracy (average correlation 0.53), this method was followed by the stratified analysis (average correlation of 0.48, that is, about 10% less, in the scale of correlation, than the interaction model), and the worst performing method was the across-environment analysis (this method had an average correlation in CV2 of 0.38, that is about 27% less than the interaction model).

**Table 6:** Estimated prediction accuracy: correlation between predicted and adjusted grain yield, averaged over 50 TRN-TST partitions), cycle 2010−2011 (W1), CV1

Model/Environments*^a^*		Correlation		Change%*^b^*	Number*^c^*
Single environment					
0iBN		0.530(0.039)		−	−
2iBN		0.629(0.038)		−	−
5iBNZ		0.472(0.054)		−	−
5iFN		0.486(0.050)		−	−
Pairs of environments		Interaction Model	Across-Env Model		
0iBN	Cor = 0.53	0.529(0.038)	0.523(0.042)	−0.3%; 1.1%	21; 33
2iBN		0.619(0.039)	0.593(0.041)	−1.6%; 4.3%	12; 49
0iBN	Cor = 0.25	0.527(0.041)	0.467(0.045)	−0.6%; 12.8%	19; 50
5iBNZ		0.468(0.053)	0.375(0.052)	−1%; 24.8%	8; 49
0iBN	Cor = 0.26	0.534(0.038)	0.494(0.044)	0.7%; 8%	40; 48
5iFN		0.485(0.050)	0.431(0.050)	−0.2%; 12.5%	26; 49
2iBN	Cor = 0.34	0.633(0.039)	0.576(0.045)	0.8%; 10%	37; 50
5iBNZ		0.477(0.050)	0.428(0.047)	0.9%; 11.2%	29; 46
2iBN	Cor = 0.33	0.625(0.041)	0.555(0.044)	−0.6%; 12.5%	18; 50
5iFN		0.479(0.051)	0.422(0.056)	−1.4%; 13.5%	15; 49
5iBNZ	Cor = 0.22	0.466(0.053)	0.406(0.056)	−1.3%; 14.8%	15; 50
5iFN		0.483(0.051)	0.448(0.053)	−0.6%; 8%	18; 48
All environments					
0iBN		0.530(0.041)	0.483(0.048)	0%; 9.7%	24; 49
2iBN		0.625(0.042)	0.558(0.046)	−0.5%; 12.1%	19; 50
5iBNZ		0.462(0.050)	0.366(0.051)	−2.2%; 26.3%	13; 50
5iFN		0.470(0.049)	0.394(0.056)	−3.3%; 19.2%	9; 50

TRN-TST, training-testing.

a Environments are described by a sequence of codes: 0i, 2i, and 5i denote the number of irrigation cycles; B/F denotes whether the planting system was “bed” (B) or “flat” (F); N/H denotes whether planting date was normal (N) or late (H, simulating heat); Z indicates no tillage.

b Change in prediction accuracy of the M×E model relative to the prediction accuracy of the single-environment (before semicolon) and relative to the prediction accuracy of the across-environment model (after semicolon).

c Number of partitions (of 50) for which the M×E model gave greater accuracy than the single-environment (before semicolon) and the across-environment model (after semicolon).

**Table 7:** Estimated prediction accuracy (correlation between predicted and adjusted grain yield, averaged over 50 TRN-TST partitions), cycle 2011-2012 (W2), CV1

Models/Environments*^a^*		Correlation		Change%*^b^*	Number*^c^*
Single environment					
0iFN		0.471(0.043)		−	−
5iBH		0.425(0.056)		−	−
5iBN		0.509(0.054)		−	−
5iFN		0.451(0.055)		−	−
Pairs of environments		Interaction Model	Across-Env model		
0iFN	Cor = 0.34	0.454(0.043)	0.375(0.052)	−3.6%; 21%	7; 50
5iBH		0.409(0.054)	0.339(0.060)	−3.7%; 20.7%	7; 50
0iFN	Cor=-0.05	0.471(0.044)	0.334(0.053)	0%; 41.2%	22; 50
5iBN		0.508(0.054)	0.353(0.058)	−0.3%; 43.7%	17; 50
0iFN	Cor = 0.31	0.453(0.045)	0.340(0.058)	−3.8%; 33.3%	7; 50
5iFN		0.437(0.053)	0.345(0.056)	−2.9%; 26.9%	14; 50
5iBH	Cor = 0.33	0.427(0.055)	0.380(0.059)	0.4%; 12.3%	24; 47
5iBN		0.507(0.057)	0.455(0.066)	−0.4%; 11.5%	23; 49
5iBH	Cor = 0.41	0.420(0.055)	0.380(0.061)	−1.2%; 10.3%	17; 47
5iFN		0.446(0.057)	0.411(0.058)	−1%; 8.5%	21; 48
5iBN	Cor = 0.41	0.500(0.054)	0.477(0.060)	−1.8%; 4.9%	18; 45
5iFN		0.449(0.059)	0.438(0.058)	−0.4%; 2.5%	23; 39
All environments					
0iFN		0.438(0.044)	0.234(0.060)	−7%; 87.7%	4; 50
5iBH		0.413(0.054)	0.356(0.067)	−2.7%; 16.2%	16; 47
5iBN		0.489(0.055)	0.386(0.065)	−4.1%; 26.6%	10; 50
5iFN		0.442(0.057)	0.396(0.059)	−1.9%; 11.6%	14; 50

TRN-TST, training-testing.

a Environments are described by a sequence of codes: 0i, 2i, and 5i denote the number of irrigation cycles; B/F denotes whether the planting system was “bed” (B) or “flat” (F); N/H denotes whether planting date was normal (N) or late (H, simulating heat); Z indicates no tillage.

b Change in prediction accuracy of the M×E model relative to the prediction accuracy of the single-environment (before semicolon) and relative to the prediction accuracy of the across-environment model (after semicolon).

c Number of partitions (of 50) for which the M×E model gave greater accuracy than the single-environment (before semicolon) and he across-environment model (after semicolon).

**Table 8:** Estimated prediction accuracy (correlation between predicted and adjusted grain yield, averaged over 50 TRN-TST partitions): cycle 2012−2013 (W3), CV1

Models/Environments*^a^*		Correlation		Change%*^b^*	Number*^c^*
Single environment					
0iFN		0.561(0.035)		−	−
2iBN		0.445(0.051)		−	−
5iBH		0.628(0.037)		−	−
5iBN		0.360(0.046)		−	−
5iFN		0.312(0.055)		−	−
Pairs of environments		Interaction Model	Across-Env Model		
0iFN	Cor = 0.17	0.559(0.036)	0.411(0.049)	−0.3%; 36.1%	18; 50
2iBN		0.445(0.051)	0.317(0.060)	0%; 40.4%	29; 49
0iFN	Cor = 0.30	0.563(0.036)	0.466(0.042)	0.2%; 20.7%	34; 50
5iBH		0.626(0.037)	0.552(0.039)	−0.3%; 13.4%	9; 50
0iFN	Cor=-0.10	0.559(0.036)	0.356(0.048)	−0.5%; 57.1%	13; 50
5iBN		0.360(0.046)	0.154(0.051)	−0.1%; 134.2%	27; 50
0iFN	Cor=-0.01	0.556(0.036)	0.400(0.045)	−0.9%; 39%	8; 50
5iFN		0.311(0.054)	0.136(0.057)	−0.5%; 129.5%	20; 50
2iBN	Cor=-0.03	0.441(0.052)	0.236(0.049)	−0.7%; 87.2%	11; 50
5iBH		0.625(0.037)	0.439(0.052)	−0.4%; 42.3%	9; 50
2iBN	Cor = 0.12	0.444(0.051)	0.370(0.049)	−0.1%; 20.2%	27; 49
5iBN		0.361(0.046)	0.300(0.047)	0.2%; 20.2%	31; 48
2iBN	Cor = 0.04	0.446(0.050)	0.345(0.052)	0.3%; 29.4%	31; 50
5iFN		0.313(0.054)	0.180(0.056)	0.1%; 73.7%	24; 50
5iBH	Cor=-0.09	0.627(0.038)	0.451(0.051)	−0.2%; 38.9%	14; 50
5iBN		0.358(0.046)	0.130(0.054)	−0.6%; 174.4%	20; 50
5iBH	Cor = 0.02	0.625(0.038)	0.485(0.046)	−0.4%; 28.8%	10; 50
5iFN		0.305(0.053)	0.125(0.060)	−2.2%; 144.1%	7; 50
5iBN	Cor = 0.55	0.352(0.046)	0.316(0.052)	−2.2%; 11.5%	15; 46
5iFN		0.309(0.053)	0.286(0.054)	−1.2%; 7.9%	19; 42
All environments					
0iFN		0.560(0.037)	0.311(0.049)	−0.2%; 80.2%	21; 50
2iBN		0.443(0.051)	0.209(0.059)	−0.4%; 112%	21; 50
5iBH		0.620(0.039)	0.333(0.059)	−1.3%; 86.1%	0; 50
5iBN		0.357(0.046)	0.156(0.048)	−0.7%; 129.4%	16; 50
5iFN		0.309(0.052)	0.111(0.064)	−1.2%; 179.2%	15; 50

TRN-TST, training-testing.

a Environments are described by a sequence of codes: 0i, 2i, and 5i denote the number of irrigation cycles; B/F denotes whether the planting system was “bed” (B) or “flat” (F); N/H denotes whether planting date was normal (N) or late (H, simulating heat); Z indicates no tillage.

b Change in prediction accuracy of the M×E model relative to the prediction accuracy of the single-environment (before semicolon) and relative to the prediction accuracy of the across-environment model (after semicolon).

c Number of partitions (out of 50) for which the M×E model gave greater accuracy than the single-environment (before semicolon) and the across-environment model (after semicolon).

**Table 9:** Estimated prediction accuracy: correlation between predicted and adjusted grain yield, averaged over 50 TRN-TST partitions, cycle 2010−2011(W1), CV2

Model/Environments*^a^*		Correlation (SE)		Change%*^b^*	Number*^c^*
Single environment					
0iBN		0.529(0.044)		−	−
2iBN		0.622(0.045)		−	−
5iBNZ		0.452(0.051)		−	−
5iFN		0.493(0.046)		−	−
Pairs of environments		Interaction Model	Across-Env Model		
0iBN	Cor = 0.53	0.599(0.036)	0.590(0.040)	13.3%; 1.5%	50; 36
2iBN		0.687(0.034)	0.664(0.037)	10.5%; 3.5%	50; 47
0iBN	Cor = 0.25	0.547(0.040)	0.476(0.049)	3.5%; 14.9%	49; 50
5iBNZ		0.467(0.050)	0.380(0.051)	3.4%; 23.1%	48; 49
0iBN	Cor = 0.26	0.544(0.038)	0.486(0.042)	3.0%; 12.0%	46; 48
5iFN		0.501(0.044)	0.452(0.048)	1.7%; 10.8%	45; 48
2iBN	Cor = 0.34	0.661(0.037)	0.587(0.042)	6.3%; 12.6%	50; 50
5iBNZ		0.496(0.044)	0.445(0.040)	9.8%; 11.4%	49; 46
2iBN	Cor = 0.33	0.648(0.043)	0.556(0.057)	4.2%; 16.5%	44; 50
5iFN		0.507(0.047)	0.456(0.054)	2.8%; 11.3%	47; 49
5iBNZ	Cor = 0.22	0.476(0.052)	0.406(0.057)	5.4%; 17.2%	33; 50
5iFN		0.491(0.047)	0.450(0.062)	−0.4%; 9.2%	35; 44
All environments					
0iBN		0.591(0.040)	0.531(0.047)	11.8%; 11.3%	50; 50
2iBN		0.697(0.033)	0.645(0.034)	12.1%; 8.2%	50; 50
5iBNZ		0.505(0.047)	0.399(0.050)	11.9%; 26.8%	50; 50
5iFN		0.516(0.051)	0.429(0.059)	4.7%; 20.4%	41; 50

TRN-TST, training-testing.

a Environments are described by a sequence of codes: 0i, 2i, and 5i denote the number of irrigation cycles; B/F denotes whether the planting system was “bed” (B) or “flat” (F); N/H denotes whether planting date was normal (N) or late (H, simulating heat); Z indicates no tillage.

b Change in prediction accuracy of the M×E model relative to the prediction accuracy of the single-environment (before semicolon) and relative to the prediction accuracy of the across-environment model (after semicolon).

c Number of partitions (of 50) for which the M×E model gave higher accuracy than the single-environment (before semicolon) and the across-environment model (after semicolon).

**Table 10:** Estimated prediction accuracy (correlation between predicted and adjusted grain yield, averaged over 50 TRN-TST partitions, cycle 2011-2012 (W2), CV2

Models/Environments*^a^*		Correlation (SE)		Change%*^b^*	Number*^c^*
Single environment					
0iFN		0.473(0.052)		−	−
5iBH		0.414(0.063)		−	−
5iBN		0.510(0.052)		−	−
5iFN		0.448(0.054)		−	−
Pairs of environments		Interaction Model	Across-Env Model		
0iFN	Cor = 0.34	0.512(0.042)	0.436(0.052)	8.3%; 17.5%	50; 47
5iBH		0.451(0.056)	0.386(0.054)	8.9%; 16.8%	50; 48
0iFN	Cor=-0.05	0.467(0.043)	0.271(0.060)	−1.2%; 72.2%	13; 50
5iBN		0.502(0.048)	0.279(0.056)	−1.6%; 80.0%	6; 50
0iFN	Cor = 0.31	0.520(0.043)	0.406(0.056)	9.8%; 27.9%	50; 49
5iFN		0.502(0.050)	0.412(0.060)	12.0%; 21.9%	50; 49
5iBH	Cor = 0.33	0.477(0.053)	0.426(0.061)	15.2%; 11.8%	48; 47
5iBN		0.546(0.047)	0.483(0.051)	7.2%; 13.2%	47; 49
5iBH	Cor = 0.41	0.500(0.049)	0.465(0.050)	20.9%; 7.7%	50; 45
5iFN		0.520(0.051)	0.490(0.055)	16.0%; 6.1%	50; 40
5iBN	Cor = 0.41	0.558(0.050)	0.541(0.052)	9.6%; 3.2%	49; 43
5iFN		0.501(0.052)	0.490(0.055)	11.8%; 2.2%	50; 37
All environments					
0iFN		0.513(0.044)	0.298(0.051)	8.5%; 72.6%	45; 50
5iBH		0.536(0.050)	0.481(0.047)	29.6%; 11.5%	50; 49
5iBN		0.531(0.044)	0.401(0.044)	4.1%; 32.4%	37; 50
5iFN		0.561(0.046)	0.523(0.049)	25.3%; 7.4%	50; 43

TRN-TST, training-testing.

a Environments are described by a sequence of codes: 0i, 2i, and 5i denote the number of irrigation cycles; B/F denotes whether the planting system was “bed” (B) or “flat” (F); N/H denotes whether planting date was normal (N) or late (H, simulating heat); Z indicates no tillage.

b Change in prediction accuracy of the M×E model relative to the prediction accuracy of the single-environment (before semicolon) and relative to the prediction accuracy of the across-environment model (after semicolon).

c Number of partitions (of 50) for which the M×E model gave greater accuracy than the single-environment (before semicolon) and the across-environment model (after semicolon).

**Table 11:** Estimated prediction accuracy (correlation between predicted and adjusted grain yield, averaged over 50 TRN-TST partitions, cycle 2012-2013, (W3), CV2

Models/Environments*^a^*		Correlation (SE)		Change%*^b^*	Number*^c^*
Single environment					
0iFN		0.559(0.036)		−	−
2iBN		0.448(0.045)		−	−
5iBH		0.630(0.035)		−	−
5iBN		0.356(0.060)		−	−
5iFN		0.307(0.042)		−	−
Pairs of environments		Interaction Model	Across-Env Model		
0iFN	Cor = 0.17	0.573(0.037)	0.356(0.053)	2.4%; 61.2%	42; 50
2iBN		0.465(0.040)	0.294(0.051)	4.0%; 58.3%	48; 50
0iFN	Cor = 0.30	0.581(0.035)	0.461(0.045)	3.8%; 25.9%	46; 50
5iBH		0.645(0.037)	0.512(0.044)	2.4%; 26.1%	37; 50
0iFN	Cor=-0.10	0.553(0.037)	0.233(0.069)	−1.2%; 136.7%	2; 50
5iBN		0.347(0.043)	0.058(0.052)	−2.4%; 498.2%	8; 50
0iFN	Cor=-0.01	0.555(0.035)	0.296(0.058)	−0.8%; 87.5%	7; 50
5iFN		0.302(0.042)	0.087(0.060)	−1.8%; 247.4%	26; 50
2iBN	Cor=-0.03	0.444(0.051)	0.180(0.047)	−0.8%; 146.8%	27; 50
5iBH		0.633(0.037)	0.337(0.052)	0.4%; 87.6%	13; 50
2iBN	Cor = 0.12	0.446(0.048)	0.321(0.047)	−0.3%; 39.2%	25; 50
5iBN		0.361(0.043)	0.279(0.048)	1.3%; 29.5%	31; 49
2iBN	Cor = 0.04	0.446(0.049)	0.255(0.061)	−0.4%; 74.6%	31; 50
5iFN		0.307(0.045)	0.142(0.057)	−0.2%; 115.3%	31; 50
5iBH	Cor=-0.09	0.623(0.038)	0.321(0.074)	−1.1%; 94.1%	3; 50
5iBN		0.345(0.045)	0.037(0.058)	−2.9%; 841.3%	9; 50
5iBH	Cor = 0.02	0.627(0.038)	0.380(0.066)	−0.5%; 65.1%	24; 50
5iFN		0.305(0.043)	0.106(0.059)	−0.6%; 188.3%	29; 50
5iBN	Cor = 0.55	0.609(0.050)	0.570(0.055)	71.2%; 7.0%	50; 49
5iFN		0.582(0.037)	0.542(0.043)	89.3%; 7.3%	50; 49
All environments					
0iFN		0.575(0.034)	0.301(0.057)	2.7%; 90.9%	50; 50
2iBN		0.466(0.043)	0.217(0.052)	4.1%; 114.7%	43; 50
5iBH		0.629(0.035)	0.281(0.046)	−0.2%; 124.3%	24; 50
5iBN		0.402(0.055)	0.243(0.054)	12.9%; 65.3%	50; 50
5iFN		0.376(0.041)	0.244(0.055)	22.3%; 53.8%	50; 49

TRN-TST, training-testing.

a Environments are described by a sequence of codes: 0i, 2i, and 5i denote the number of irrigation cycles; B/F denotes whether the planting system was “bed” (B) or “flat” (F); N/H denotes whether planting date was normal (N) or late (H, simulating heat); Z indicates no tillage.

b Change in prediction accuracy of the M×E model relative to the prediction accuracy of the single-environment (before semicolon) and relative to the prediction accuracy of the across-environment model (after semicolon).

c Number of partitions (out of 50) for which the M×E model gave greater accuracy than the single-environment (before semicolon) and the across-environment model (after semicolon).

#### Stratified analysis:

The within-environment analysis yielded prediction correlations ranging from moderately low (0.307 for environment 5iFN in W3) to moderately high (0.630 for environment 5iBH in W3).

#### Across-environment analysis:

Overall, this method was the worst performing one. In CV1 the across-environment analysis performed worse than the stratified analysis and the interaction model; this in every environment and dataset. In CV2, the joint analysis of data from different environments ignoring G×E performed worse than the stratified analysis when the pairs of environments analyzed together had a correlation lower than 0.3; however, the across-environment model tended to outperform the stratified analysis whenever the correlation between environments was larger than 0.4. On the other hand, the across-environment model was systematically outperformed by the M×E model both in CV1 and in CV2. In CV2, the difference in prediction accuracy between these two methods was low when the pairs of environments analyzed were positively correlated (*e.g.*, in W1, environments 0iBN and 2iBN; see Table 9) and large, and in favor of the M×E model, when the set of environments analyzed were negatively correlated (*e.g.*, in W3, in the joint analysis of 0iFN and 5iBN).

#### M×E model:

As previously stated, in CV1, the M×E model performed similarly to the stratified analysis. However, in CV2, the joint analysis of all environments using the M×E model gave, relative to the stratified analysis, average gains in prediction accuracy ranging from 4.7 to 12.1% in W1, 4.1 to 29.6% in W2 and −0.2 to 22.3% in W3. The patterns of gain/loss in prediction accuracy achieved in CV2 with the M×E model, relative to the stratified analysis, were directly related to the correlation between environments and to the proportion of genomic variance explained by the main effects. Environments exhibiting positive correlations with other environments benefited greatly from the use of multienvironment models (*e.g.*, 0iBN or 2iBN in W1, 5iBH and 5iFN in W2, and 5iFN and 5iBN in W3). For these environments in all 50 partitions, the multienvironment model fitted to all environments jointly had higher prediction accuracy than the within-environment model (Figure 2).

In CV2, the joint analysis of all environments using a M×E model gave, in general, a greater prediction accuracy than the one achieved with analyses of pairs of environments; this was particularly clear in W1 and W2 (Table 9 and Table 10, respectively), with the only exception of 5iBN in W2, where the average prediction accuracy was slightly higher in the bivariate analysis than in the multienvironment model applied to all environments jointly. The situation in W3 was slightly different; here, the predictive performance of analyses based on pairs of environments was similar to that of the joint multienvironment analysis of all conditions (Table 11).

## Discussion

Several studies have documented the benefits of using multienvironment models, relative to single-environment analysis (Burgueño *et al.* 2012; Dawson *et al.* 2013; Jarquin *et al.* 2014). Multienvironment analysis can model G×E interactions using covariance functions (Burgueño *et al.* 2012), markers and environmental covariates (Jarquin *et al.* 2014; Heslot *et al.* 2014) ,or by modeling M×E interactions. In this article, we adapted this approach, previously used in QTL models by Moreau *et al.*(2004), Van Eeuwijk *et al.* (2005), Boer *et al.* (2007), and Malosetti *et al.* (2004, 2008), to whole-genome regression models where phenotypes were regressed on large numbers of genome-wide markers.

Relative to the standard multienvironment models with structured or unstructured covariances such as those used by Burgueño *et al.* (2012), the M×E model has advantages and disadvantages. On one hand, the interaction model: (i) is easy to implement using existing software for GS; (ii) leads to a decomposition of marker effects into components that are stable across environments (main effects) and environment-specific deviations (interactions) that can shed light on which genomic regions are most responsible for G×E; and (iii) can be implemented with any of the priors commonly used in GS, including not only shrinkage methods such as the GBLUP, but also variable selection methods. On the other hand, the M×E model imposes restrictions on co-variance patterns: the covariance between environments is forced to be positive and constant across pairs of environments. Therefore, the M×E model is best suited for the joint analysis of sets of environments that are positively and similarly correlated. In those cases, the parsimony of the M×E model can be advantageous; however, the pattern may be too restrictive in cases where the genomic variance cannot be approximated with the structure imposed by the interaction model (Meyer and Kirkpatrick 2008).

Among the three data sets considered here, two of them, (W1 and W2) had patterns of phenotypic (sample) correlations with moderately positive correlations between pairs of environments, while the third one (W3) exhibited a great deal of G×E, with some pairs of environments exhibiting either null or negative sample phenotypic correlations. The environments included in our data sets were managed field conditions aiming to approximate three basic target sets of environments (mega-environments) comprising three main agroclimatic regions previously defined and widely used by CIMMYT’s Global Wheat Breeding Program (Braun *et al.* 1996). These three agroclimatic regions are represented by megaenvironment 1 (low rainfall and irrigated), megaenvironment 4 (drought), and megaenvironment 5 (heat). Results indicated that environments clustered more along the lines of full irrigation, drought, and heat than based on whether they include beds or flat planting systems, and zero or conventional tillage.

### Variance components

**Figure 3:**
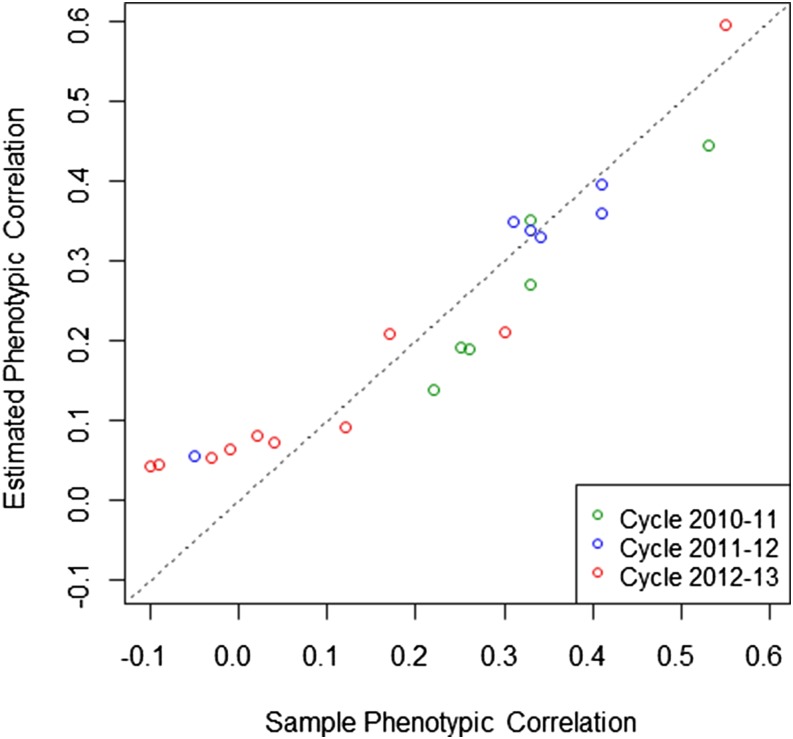
Estimated phenotypic correlation (based on estimates of variance components) *vs.* sample phenotypic correlation. The estimated phenotypic correlation was obtained from estimates of variance components (see Table 3, Table 4, and Table 5) from the bivariate analysis.

The M×E model fitted the data much better than the across-environment model that ignored G×E. Furthermore, the estimates of variance components from the M×E model indicated that the proportion of genomic variance explained by the main effect of markers is directly related to the (sample empirical) phenotypic correlation between environments. Analytically, under the assumptions of the M×E model described and used in this article, if $y_{1i}=\mu_{1}+u_{0i}+u_{1i}+\varepsilon_{1i}$ and $y_{2i}=\mu_{2}+u_{0i}+u_{2i}+\varepsilon_{2i}$ are the equations for the phenotype of the *i^th^* line in environments *1* and *2*, respectively, then the phenotypic correlation can be expressed as a function of variance components, indeed $Cor\left(y_{1i},y_{2i}\right)=\frac{Cov\left(y_{1i},y_{2i}\right)}{\sqrt{Var\left(y_{1i}\right)Var\left(y_{2i}\right)}}=\frac{\sigma_{u0}^{2}}{\sqrt{\sigma_{u0}^{2}+\sigma_{u1}^{2}+\sigma_{\varepsilon }^{2}}\sqrt{\sigma_{u0}^{2}+\sigma_{u2}^{2}+\sigma_{\varepsilon }^{2}}}$. Figure 3 displays the estimated phenotypic correlations derived using estimates of variance components obtained from analyses based on pairs of environments (see Table 3, Table 4, and Table 5) *vs.* the observed sample phenotypic correlations. Overall, the estimated phenotypic correlation based on variance components was linearly related to the sample phenotypic correlation for pairs of environments having positive sample phenotypic correlations. However, as the sample phenotypic correlation approached zero or became negative, the relationship flattened out. This happens because, in the interaction model, the covariance is represented by the variance of the main effects and, therefore, it is bound to be non-negative.

### Prediction accuracy

The prediction analysis conducted in this study yielded levels of accuracy (correlation between phenotypes and predicted genomic values) that are consistent with previous reports for grain yield prediction accuracy using single- and multi-environment models (Burgueño *et al.* 2012). Overall, the interaction model was either the best performing method (CV2) or performed close to the best performing method (in CV1 the stratified analysis and the interaction model performed similarly). These results are consistent with those of previous studies (*e.g.*, Burgueño *et al.* 2012; Jarquin *et al.* 2014) that have used similar validation designs (CV1 and CV2) and have reported similar predictive performance of the stratified and multivariate analysis in CV1, and clear superiority of the multivariate approach in CV2. Additionally, we also considered an across-environment analysis that ignores G×E. In our study this approach was clearly the worst-performing one; this finding highlights the importance of considering G×E when analyzing multi-environment data.

The gains in prediction accuracy obtained in CV2 with the M×E model were directly related to the correlations between environments. Considering environments that had positive phenotypic correlations among them, the use of the M×E model yielded in CV2 gains, relative to the stratified analysis, that were either moderate (on the order of 5%) or very substantial (on the order of 29%). In CV2 the only cases where the stratified analysis was better than the interaction model are those based on the joint analysis of pairs of environments that had close to null or negative correlations (*e.g.*, 5iBH in W3). This happened because, as discussed previously, the interaction model forces the covariance between environments to be non-negative.

In prediction problems such as that of CV2, the superiority of the M×E relative to the stratified analysis can be attributed to the fact that the M×E model allows borrowing of information within line across environments, that is: when deriving predictions for a given line, the M×E model benefits from records from the same line collected in correlated environments (this borrowing of information also happens in the across-environment GBLUP; however, in the across-environment GBLUP borrowing of information within line across environments is achieved at the expense of forcing the effects to be constant across environments). This feature of the M×E model can be exploited in prediction problems such as CV2; however, such borrowing of information within line is not possible in CV1 and, consequently, the M×E model performs similarly to the stratified analysis for prediction of performance of lines that have no phenotypic records.

### How many environments?

The interaction model can be applied to all available environments or to other sets (*e.g.*, pairs) of environments. For data sets such as W1, where all environments showed positive correlations of similar magnitude, the joint analysis of all environments was clearly superior to analyses based on pairs of environments. However, in data sets exhibiting complex co-variance patterns (such as W3), joint analysis of all environments using an interaction model imposes inadequate restrictions on co-variance patterns and, consequently, bivariate analysis seems more appropriate.

### Extensions

In our study, because of the limitations of the software used, we implemented the M×E model in which the error variance was assumed to be homogeneous across environments. In principle, the model can be easily extended to accommodate environment-specific variances.

In this study, we presented and applied the interaction model using Gaussian priors. We did this because the GBS marker data contain a large proportion of missing values. However, with high-density panels of high-quality markers (*e.g.*, single-nucleotide polymorphisms) it would make perfect sense to use other priors. For instance, it could be used with priors that induce differential shrinkage of estimates or variable selection (de los Campos *et al.* 2013); such treatment would potentially aid in identifying sets of markers with effects that are stable across environments and others that are responsible for G×E.

The M×E model presented in this article is an easy-to-implement and easy-to-interpret approach for modeling G×E in genomic models. The model allows decomposing marker effects and genomic variance into components that are stable across environments (main effects) and components that are environment-specific (interaction terms). The model can be implemented easily using existing software for GS. Predictions from the interaction model had either similar (CV1) or greater (CV2) accuracy than the single-environment analysis and were always more accurate than those derived from an across-environment analysis that ignored G×E; therefore, the proposed model should be useful for selection based on either stability (main effects only) or for target environments (based on total genomic value). The interaction model is not free of limitations; in particular, it is important to note that the genetic covariance between any pair of environments is represented by the variance of the main effect; therefore, it is restricted to being positive and the same for all pairs of environments analyzed jointly. Therefore, the model should be more effective when applied to subsets of environments that have positive and similar correlations.

## 

## Supplementary Material

Supporting Information

## References

[bib1] Boer M. P. Wright D. Feng L. Podlich D. W. Luo L. 2007 A mixed-model quantitative trait loci (QTL) analysis for multiple-environment trial data using environmental covariables for QTL-by-environment interactions, with an example in maize. Genetics 177: 1801–1813. 17947443 10.1534/genetics.107.071068 PMC2147942

[bib2] Braun H. J. Rajaram S. van Ginkel M., 1996 CIMMYT’s approach to breeding for wide adaptation. Euphytica 92: 175–183.

[bib3] Burgueño J. Crossa J. Cornelius P. L. Trethowan R. McLaren G. 2007 Modeling additive × environment and additive × additive × environment using genetic covariance of relatives of wheat genotypes. Crop Sci. 47: 311–320.

[bib4] Burgueño J. de los Campos G. Weigel K. Crossa J., 2012 Genomic prediction of breeding values when modeling genotype × environment interaction using pedigree and dense molecular markers. Crop Sci. 52: 707–719.

[bib5] Crossa J. Burgueño J. Cornelius P. L. Trethowan R. Krishnamachari A., 2006 Modeling genotype × environment interaction using additive genetic covariances of relatives for predicting breeding values of wheat genotypes. Crop Sci. 46: 1722–1733.

[bib6] Crossa J. de los Campos G. Pérez P. Gianola D. Burgueño J. 2010 Prediction of genetic values of quantitative traits in plant breeding using pedigree and molecular markers. Genetics 186: 713–724. 20813882 10.1534/genetics.110.118521 PMC2954475

[bib7] Crossa J. Pérez P. de los Campos G. Mahuku G. Dreisigacker S. 2011 Genomic selection and prediction in plant breeding. J. Crop Improv. 25: 239–261.

[bib8] Dawson J. C. Endelman J. B. Heslot N. Crossa J. Poland J. 2013 The use of unbalanced historical data for genomic selection in an international wheat breeding program. Field Crops Res. 154: 12–22.

[bib9] de los Campos, G., and P. Pérez-Rodriguez, 2014 Bayesian generalized linear regression. R package version 1.0.1. Available at: http://CRAN.R-project.org/package=BGLR. Accessed February 17, 2015.

[bib10] de los Campos G. Naya H. Gianola D. Crossa J. Legarra A. 2009 Predicting quantitative traits with regression models for dense molecular markers and pedigree. Genetics 182: 375–385. 19293140 10.1534/genetics.109.101501 PMC2674834

[bib11] de los Campos G. Gianola D. Rosa G. J. M. Weigel K. Crossa J., 2010 Semi-parametric genomic-enabled prediction of genetic values using reproducing kernel Hilbert spaces methods. Genet. Res. 92: 295–308. 10.1017/S0016672310000285 20943010

[bib12] de los Campos G. Hickey J. M. Pong-Wong R. Daetwyler H. D. Calus M. P. L., 2013 Whole-genome regression and prediction methods applied to plant and animal breeding. Genetics 193: 327–345. 22745228 10.1534/genetics.112.143313 PMC3567727

[bib13] Eberhart S. A. Russell W. A., 1966 Stability parameters for comparing varieties. Crop Sci. 6: 36–40.

[bib14] Finlay K. W. Wilkinson G. N., 1963 The analysis of adaptation in a plant breeding program. Aust. J. Agric. Res. 14: 742–754.

[bib15] Heslot N. Yang H. P. Sorrells M. E. Jannink J. L., 2012 Genomic selection in plant breeding: a comparison of models. Crop Sci. 52: 146–160.

[bib16] Heslot N. Akdemir D. Sorrells M. E. Jannink J. L., 2014 Integrating environmental covariates and crop modeling into the genomic selection framework to predict genotype by environment interactions. Theor. Appl. Genet. 127: 463–480. 24264761 10.1007/s00122-013-2231-5

[bib17] Jarquin D. Crossa J. Lacaze X. Cheyron P. D. Daucourt J. 2014 A reaction norm model for genomic selection using high-dimensional genomic and environmental data. Theor. Appl. Genet. 127: 595–607. 24337101 10.1007/s00122-013-2243-1 PMC3931944

[bib18] Malosetti M. Voltas J. Romagosa I. Ullrich S. E. van Eeuwijk F. A., 2004 Mixed models including environmental covariables for studying QTL by environment interaction. Euphytica 137: 139–145.

[bib19] Malosetti M. Ribaut J. M. Vargas M. Crossa J. van Eeuwijk F. A., 2008 A multi-trait multi-environment QTL mixed model with an application to drought and nitrogen stress trials in maize (*Zea mays* L.). Euphytica 161: 241–257.

[bib20] Meuwissen T. H. E. Hayes B. J. Goddard M. E., 2001 Prediction of total genetic value using genome-wide dense marker maps. Genetics 157: 1819–1829. 11290733 10.1093/genetics/157.4.1819 PMC1461589

[bib21] Meyer K. Kirkpatrick M., 2008 Perils of parsimony: properties of reduced-rank estimates of genetic covariance matrix. Genetics 180: 1153–1166. 18757923 10.1534/genetics.108.090159 PMC2567364

[bib22] Moreau L. Charcosset A. Gallais A., 2004 Use of trial clustering to study QTL x environment effects for grain yield and related traits in maize. Theor. Appl. Genet. 110: 92–105. 15551040 10.1007/s00122-004-1781-y

[bib23] Pérez-Rodriguez P. Gianola D. Gonzalez-Camacho J. M. Crossa J. Manes Y. 2012 A comparison between linear and non-parametric regression models for genome-enabled prediction in wheat. G3(Bethesda) 2: 1595–1605. 23275882 10.1534/g3.112.003665 PMC3516481

[bib24] Piepho H. P., 1997 Analyzing genotype-environment data by mixed models with multiplicative effects. Biometrics 53: 761–766.

[bib25] Piepho H. P., 1998 Empirical best linear unbiased prediction in cultivar trials using factor analytic variance covariance structure. Theor. Appl. Genet. 97: 195–201.

[bib26] Poland J. Endelman J. Dawson J. Ruthkoski J. Wu S. 2012 Genomic selection in wheat breeding using genotyping-by-sequencing. The Plant Genome 5: 103–113.

[bib27] R Core Team, 2013 R: A Language and Environment for Statistical Computing. Vienna, Austria: R Foundation for Statistical Computing. Available at: http://www.R-project.org. Accessed February 17, 2015.

[bib28] Smith A. B. Cullis B. R. Thompson R., 2005 The analysis of crop cultivar breeding and evaluation trials: an overview of current mixed model approaches. J. Agric. Sci. 143: 449–462.

[bib29] van Eeuwijk F. A. Malosetti M. Yin X. Struik P. C. Stam P., 2005 Statistical models for genotype by environment data: from conventional ANOVA models to eco-physiological QTL models. Aust. J. Agric. Res. 56: 883–894.

[bib30] VanRaden P. M., 2007 Genomic measures of relationship and inbreeding. Interbull Bull 37: 33–36.

[bib31] VanRaden P. M., 2008 Efficient methods to compute genomic predictions. J. Dairy Sci. 91: 4414–4423. 18946147 10.3168/jds.2007-0980

[bib32] Yates F. Cochran W. G., 1938 The analysis of groups of experiments. J. Agric. Sci. 28: 556–580.

