# Supplementary material for: Increased Prediction Accuracy in Wheat Breeding Trials Using a Marker × Environment Interaction Genomic Selection Model
Source: G3 (Bethesda). 2015 Feb 6;5(4):569–82. doi: 10.1534/g3.114.016097 (PMC4390573; doi:10.1534/g3.114.016097)
Supplement: Supporting Information [file supp_5_4_569__index.html]

Increased Prediction Accuracy in Wheat Breeding Trials Using a Marker × Environment Interaction Genomic Selection Model — Supporting Information 

# Increased Prediction Accuracy in Wheat Breeding Trials Using a Marker × Environment Interaction Genomic Selection Model

## Supporting Information for Lopez-Cruz *et al.*, 2015

**Files in this Data Supplement:**

- File S4 - Supporting Information (PDF, 965 KB)
- File S1 - Phenotypic and genotypic information on a data set used in this study (45th International Bread Wheat Screening Nurseries, IBWSN). (.RData, 3 MB)
- File S2 - Phenotypic and genotypic information on a data set used in this study (46th International Bread Wheat Screening Nurseries, IBWSN). (.RData, 3 MB)
- File S3 - Phenotypic and genotypic information on a data set used in this study (47th International Bread Wheat Screening Nurseries, IBWSN). (.RData, 4 MB)
