# Supplementary material for: Increased Prediction Accuracy in Wheat Breeding Trials Using a Marker × Environment Interaction Genomic Selection Model
Source: G3 (Bethesda). 2015 Feb 6;5(4):569–82. doi: 10.1534/g3.114.016097 (PMC4390573; doi:10.1534/g3.114.016097)
Supplement: Supporting Information [file supp_g3.114.016097_FileS4.pdf]

Supporting Information for:

Increased prediction accuracy in wheat breeding lines using a marker × environment interaction genomic selection model

by

Marco Lopez-Cruz*, Jose Crossa*, David Bonnett*, Susanne Dreisigacker*, Jesse Poland[§], Jean-Luc Jannink[†], Ravi P. Singh*, Enrique Autrique* and Gustavo de los Campos[‡,1]

1. Supplementary data

The following files are provided:

File S1: Wheat_GY_45IBWSN_standarized_data.RData

File S2: Wheat_GY_46IBWSN_standarized_data.RData

File S3: Wheat_GY_47IBWSN_standarized_data.RData

Each of these files contains the following R-objects:

- Y (n×s) a numeric matrix with centered and standarized yield records. Each column represent records taken in a particular environment. The column-names of Y, `colnames(Y)`, gives the codes that identify the environments and the row-names of Y, `rownames(Y)`, gives the IDs of the wheat lines.
- G (n×n) a genomic relationship matrix computed based on the GBS data. The line IDs can be retrieved using either `rownames(G)` or `colnames(G)`.

2. Software and scripts for data analysis

Boxes 1a and 1b provide simplified scripts that can be used to fit the single-environment model. The example uses the third data set (File S3) as an example, but this can be modified by changing the file name in line 2 of Box 1a.

Box 1a. Within-Environment (i.e., stratified) GBLUP (model fitting)

```
1   rm(list=ls())
2   load("Wheat_GY_47IBWSN_standarized_data.RData")
3   library('BGLR')
4
5   env <- 4 # choose any number in 1:ncol(Y)
6   prefix <- paste(colnames(Y)[env],"_",sep="")
7
8   # Fitting the model
9   ETA <- list(G=list(K=G,model='RKHS'))
10  fm <- BGLR(y=Y[,env],ETA=ETA,nIter=12000,burnIn=2000,saveAt=prefix)
11
```

Box 1b provides code that can be used to extract estimates of variance components and predictions obtained after fitting the model in Box 1a.

Box 1b. Within-Environment (i.e., stratified) GBLUP (post-hoc)

```
# Extracting some estimates & predictions
fm$varE          # residual variance
fm$ETA[[1]]$varU # genomic variance
fm$ETA[[1]]$u    # genomic predictions

# Some trace plots
varE <- scan(paste(prefix,'varE.dat',sep=''))
plot(varE,type='o',cex=.5,col=4)

varU <- scan(paste(prefix,'ETA_1_varU.dat',sep=''))
plot(varU,type='o',cex=.5,col=4)
```

Box 2a provides code that can be used to fit an across-environment model to combined data from a set of environments. As before, the data set used is defined with the file name given in line 2. In line 5 we define the set of environments to be analyzed jointly; we analyze environments 4 and 5 jointly in the example, but this can be modified easily. For instance, if one wants to analyze all environments jointly, one can set in line 5 `env<-1:ncol(Y)`. The across-environment model includes: (i) environment-specific intercepts; this is defined in lines 12-14 in Box 2a; and (ii) the

effect of the markers, common to all environments; this is defined in lines 16-18. Finally, the model is fitted in line 21.

Box 2a. Across-Environment Model (model fitting)

```
1  rm(list=ls())
2  load("Wheat_GY_47IBWSN_standarized_data.RData")
3  library('BGLR')
4  
5  env <- c(4,5) # choose any set of environments from 1:ncol(Y)
6  
7  nEnv <- length(env)
8  prefix <- paste(c('Across',colnames(Y)[env],''),collapse='_')
9  
10 y <- as.vector(Y[,env])
11 
12 # Fixed effect (env-intercepts)
13 envID <- rep(env,each=nrow(Y))
14 ETA <- list(list(~factor(envID)-1,model="FIXED"))
15 
16 # Effects of markers
17 G0 <- kronecker(matrix(nrow=nEnv,ncol=nEnv,1),G)
18 ETA[[2]] <- list(K=G0,model='RKHS')
19 
20 # Model Fitting
21 fm <- BGLR(y=y,ETA=ETA,nIter=12000,burnIn=2000,saveAt=prefix)
22 
```

Box 2b illustrates how to extract parameter estimates and predictions and how to retrieve samples obtained after fitting the model in Box 2a.

Box 2b. Across-Environment Model (post-hoc)

```
# Extracting estimates of variance parameters
fm$varE          # residual variance
fm$ETA[[2]]$varU # genomic variance

# Predictions (this is all within training)
tmpEnv <- 1
plot(y[envID==env[tmpEnv]]~fm$yHat[envID==env[tmpEnv]])

# Samples
varE <- scan(paste(prefix,'varE.dat',sep=''))
plot(varE,type='o',cex=.5,col=4)

varU0 <- scan(paste(prefix,'ETA_2_varU.dat',sep=''))
plot(varU0,type='o',cex=.5,col=4)

```

Box 3a provides code that can be used to fit a M×E model to combined data from a set of environments. Similarly, the data set used is defined by setting the file name in line 2. In line 5 we define the set of environments to be analyzed jointly; we analyze environments 4 and 5. The M×E model includes: (i) environment-specific intercepts; this is defined in lines 12-14 in Box 3a; (ii) the main effect of the markers; this is defined in lines 16-18; and (iii) co-variance structures for M×E. These co-variance structures are created in lines 21-25. Finally, the model is fitted in line 28.

Box 3a. Marker-by-Environment Interaction Model (model fitting)

```
1   rm(list=ls())
2   load("Wheat_GY_47IBWSN_standarized_data.RData")
3   library('BGLR')
4
5   env <- c(4,5) # choose any set of environments from 1:ncol(Y)
6
7   nEnv <- length(env)
8   prefix <- paste(c('MxE',colnames(Y)[env],''),collapse='_')
9
10  y <- as.vector(Y[,env])
11
12  # Fixed effect (env-intercepts)
13  envID <- rep(env,each=nrow(Y))
14  ETA <- list(list(~factor(envID)-1,model="FIXED"))
15
16  # Main effects of markers
17  G0 <- kronecker(matrix(nrow=nEnv,ncol=nEnv,1),G)
18  ETA[[2]] <- list(K=G0,model='RKHS')
19
20  # Adding interaction terms
21  for(i in 1:nEnv){
22     tmp <- rep(0,nEnv) ; tmp[i] <- 1
23     G1 <- kronecker(diag(tmp),G)
24     ETA[[(i+2)]] <- list(K=G1, model='RKHS')
25  }
26
27  # Model Fitting
28  fm <- BGLR(y=y,ETA=ETA,nIter=12000,burnIn=2000,saveAt=prefix)
29
```

Box 3b illustrates how to extract parameter estimates and predictions and how to retrieve samples obtained after fitting the model in Box 3a.

Box 3b. Marker-by-Environment Interaction Model (post-hoc)

```
# Extracting estimates of variance parameters
fm$varE            # residual variance
fm$ETA[[2]]$varU  # genomic variance (main effect)
vGInt <- rep(NA,nEnv)
for(i in 1:nEnv){ # interaction variances
   vGInt[i] <- fm$ETA[[(i+2)]]$varU
}
vGInt

# Predictions (this is all within training)
tmpEnv <- 1
plot(y[envID==env[tmpEnv]]~fm$yHat[envID==env[tmpEnv]])

# Samples
varE <- scan(paste(prefix,'varE.dat',sep=''))
plot(varE,type='o',cex=.5,col=4)

varU0 <- scan(paste(prefix,'ETA_2_varU.dat',sep=''))
plot(varU0,type='o',cex=.5,col=4)

varU1 <- matrix(nrow=length(varU0),ncol=nEnv,NA)
for(i in 1:nEnv){
   varU1[,i] <- scan(paste(prefix,'ETA_',i+2,'_varU.dat',sep=''))
}

tmpEnv <- 1
plot(varU1[,tmpEnv],type='o',col=4,cex=.5)

```

Boxes 1a, 2a, and 3a illustrate how to fit models to the full data set. Only slight modifications of the code are needed to assess prediction accuracy of TRN-TST experiments. BGLR supports missing values in the response; therefore, to assess prediction accuracy in a testing data set, one possibility is to insert NAs in the entries in the testing data set. The following Boxex illustrates how to create a TRN-TST partition for CV1 (Box 4a) and one for CV2 (Box 4b). After runing this code, the matrices YNA has missing values for the entries corresponding to the TST set.

Box 4a. Creating a Testing Sets for CV1

```
rm(list=ls())
load("Wheat_GY_47IBWSN_standarized_data.RData")
library('BGLR')
set.seed(12345)

env <- c(4,5) # choose any set of environments from 1:ncol(Y)
nEnv <- length(env)
Y <- Y[,env]
n <- nrow(Y)

percTST<-0.3
nTST <- round(percTST*n)
tst<-sample(1:n,size=nTST,replace=FALSE)
YNA <- Y
YNA[tst,]<-NA
```

Box 4b. Creating a Testing Sets for CV2

```
rm(list=ls())
load("Wheat_GY_47IBWSN_standarized_data.RData")
library('BGLR')
set.seed(12345)

env <- c(4,5) # choose any set of environments from 1:ncol(Y)
nEnv <- length(env)
Y <- Y[,env]
n <- nrow(Y)

percTST<-0.3
nTST <- round(percTST*n)
nNA <- nEnv*nTST
if(nNA<n){ indexNA <- sample(1:n,nNA,replace=FALSE) }
if(nNA>=n){
  nRep <- floor(nNA/n)
  remain <- sample(1:n,nNA%%n,replace=FALSE)
  a0 <- sample(1:n,n,replace=FALSE)
  indexNA <- rep(a0,nRep)
  if(length(remain)>0){
         a1 <- floor(length(indexNA)/nTST)*nTST
         a2 <- nNA - a1 - length(remain)
         bb <- sample(a0[!a0%in%remain],a2,replace=FALSE)
         noInIndexNA <- c(rep(a0,nRep-1),a0[!a0%in%bb])
         indexNA <- c(noInIndexNA,bb,remain)
  }
}
indexEnv <- rep(1:nEnv,each=nTST)
YNA <- Y
for(j in 1:nEnv) YNA[indexNA[indexEnv==j],j] <- NA
```

Once the YNA matrix is created (see Box 4a for CV1-type partitions and Box 4b for CV2-type partitions), this data matrix can be used instead of the original data-matrix (Y) to fit models using the code presented in Boxes 1a, 2a and 3a. The following Box illustrates how to fit the single-environment model and a two-environment model for the set of environments selected in Box 4.

Box 5. Fitting Models to TRN-TST Partitions (continues from Box 4b)

```
## Single environments models #####################################
 YHatSE <- matrix(nrow=nrow(Y),ncol=ncol(Y),NA)
 ETA <- list(G=list(K=G,model='RKHS'))

 for(i in 1:nEnv){
   prefix <- paste(colnames(Y)[i],"_",sep="")
   fm <-BGLR(y=YNA[,i],ETA=ETA,nIter=12000,burnIn=2000,saveAt=prefix)
   YHatSE[,i] <- fm$yHat
 }

## Across environment model (ignoring GxE) #######################
 yNA <- as.vector(YNA)

 # Fixed effect (env-intercepts)
 envID <- rep(env,each=nrow(Y))
 ETA <- list(list(~factor(envID)-1,model="FIXED"))

 # Main effects of markers
 G0 <- kronecker(matrix(nrow=nEnv,ncol=nEnv,1),G)
 ETA[[2]] <- list(K=G0,model='RKHS')

 # Model Fitting
 prefix <- paste(c('Across',colnames(Y),''),collapse='_')
 fm <- BGLR(y=yNA,ETA=ETA,nIter=12000,burnIn=2000,saveAt=prefix)
 YHatAcross <- matrix(fm$yHat,ncol=nEnv)

## MxE Interaction Model #########################################
 # Adding interaction terms
 for(i in 1:nEnv){
   tmp <- rep(0,nEnv) ; tmp[i] <- 1;  G1 <- kronecker(diag(tmp),G)
   ETA[[(i+2)]] <- list(K=G1,model='RKHS')
 }

 # Model Fitting
 prefix <- paste(c('MxE',colnames(Y),''),collapse='_')
 fm <- BGLR(y=yNA,ETA=ETA,nIter=12000,burnIn=2000,saveAt=prefix)
 YHatInt <- matrix(fm$yHat,ncol=nEnv)

```

In Box 6 we illustrate how to compute the within-environment prediction accuracy in the testing data set used in Box 5. Note that the estimates reported in the article are the average of 50 TRN-TST partitions.

Box 6. Computing the within-environment correlation (continues from Box 5)

```
COR <- matrix(nrow=length(env),ncol=3,NA)
colnames(COR) <- c('SingleEnv', 'AcrossEnv', 'MxE')
rownames(COR) <- colnames(Y)

for(i in 1:nEnv){
   tst <- which(is.na(YNA[,i]))
   COR[i,1] <- cor(Y[tst,i],YHatSE[tst,i])
   COR[i,2] <- cor(Y[tst,i],YHatAcross[tst,i])
   COR[i,3] <- cor(Y[tst,i],YHatInt[tst,i])
}
COR
```
